# Immune Classification and Immune Landscape Analysis of Triple-Negative Breast Cancer

**DOI:** 10.3389/fgene.2021.710534

**Published:** 2021-11-02

**Authors:** Shaojun Hu, Xiusheng Qu, Yu Jiao, Jiahui Hu, Bo Wang

**Affiliations:** ^1^ Oncology Department, First Affiliated Hospital of Jiamusi University, Jiamusi, China; ^2^ Chemotherapy Department, First Affiliated Hospital of Jiamusi University, Jiamusi, China

**Keywords:** triple-negative breast cancer, TCGA database, immune microenvironment, immune subtype, immunotherapy

## Abstract

**Background:** To classify triple-negative breast cancer (TNBC) immunotyping using the public database, analyze the differences between subtypes in terms of clinical characteristics and explore the role and clinical significance of immune subtypes in TNBC immunotherapy.

**Methods:** We downloaded TNBC data from the cBioPortal and GEO databases. The immune genes were grouped to obtain immune gene modules and annotate their biological functions. Log-rank tests and Cox regression were used to evaluate the prognosis of immune subtypes (IS). Drug sensitivity analysis was also performed for the differences among immune subtypes in immunotherapy and chemotherapy. In addition, dimension reduction analysis based on graph learning was utilized to reveal the internal structure of the immune system and visualize the distribution of patients.

**Results:** Significant differences in prognosis were observed between subtypes (IS1, IS2, and IS3), with the best in IS3 and the worst in IS1. The sensitivity of IS3 to immunotherapy and chemotherapy was better than the other two subtypes. In addition, Immune landscape analysis found the intra-class heterogeneity of immune subtypes and further classified IS3 subtypes (IS3A and IS3B). Immune-related genes were divided into seven functional modules (The turquoise module has the worst prognosis). Five hub genes (RASSF5, CD8A, ICOS, IRF8, and CD247) were screened out as the final characteristic genes related to poor prognosis by low expression.

**Conclusions:** The immune subtypes of TNBC were significantly different in prognosis, gene mutation, immune infiltration, drug sensitivity, and heterogeneity. We validated the independent role of immune subtypes in tumor progression and immunotherapy for TNBC. This study provides a new perspective for personalized immunotherapy and the prognosis evaluation of TNBC patients in the future.

## 1 Introduction

Breast cancer (BC) has become a central women’s health problem worldwide, with the highest incidence of malignant tumors among Chinese women (Chen et al., 2018). According to the expression of estrogen receptors (ER), progesterone receptors (PGR), and human epidermal growth factor receptor 2 (HER2), three subtypes are commonly used for breast cancer clinical classification: the ER/PGR-positive subtype, the HER2-positive subtype, and TNBC, with the most aggressive behavior. TNBC accounts for nearly 15% of BC patients annually, and it is characterized by a high metastasis/recurrence rate and a short survival time (Shen et al., 2020). Approximately 50% of TNBC patients who undergo radical surgery without metastasis will develop a disease recurrence (Liedtke et al., 2008). The five-year survival rates for regional and metastatic TNBC have been reported to be 65 and 11%, respectively, and these are significantly lower than those for other tumor subtypes (Gupta et al., 2020).

Traditional treatment regimens, including surgical resection, radiotherapy, and chemotherapy, often have poor outcomes and prominent adverse effects in TNBC patients (Liedtke et al., 2008; Foulkes et al., 2010). In recent years, immunotherapy has been used with some success to treat an increasing number of cancer patients, such as those with melanoma, renal cell carcinoma, and lung cancer (Schachter et al., 2017; Topalian et al., 2019). Similarly, TNBC also has potential immunogenicity due to its genomic instability and high mutation rate (Kwa and Adams, 2018). At present, immunotherapy for BC mainly involves tumor vaccine therapy, cytokine therapy, monoclonal antibody therapy, and adoptive cell therapy. However, deficiencies, including low response rates and limited survival benefits, make it difficult for immunotherapy to be widely used in TNBC, suggesting that there may be a specific subtype associated with immunotherapy benefits (Caparica et al., 2019; Gupta et al., 2020). Therefore, a better understanding of the tumor immune microenvironment (TIME) is needed to improve immunotherapy’s response to and outcomes.

In this study, we conducted a multicohort retrospective study to identify three verifiable immune subtypes of TNBC. Then, subtype validation and comprehensive molecular identification were performed using independent data. The results suggested that each IS was associated with different gene expression profiles. Different subtypes showed widely different patterns in tumor genetic aberrations, cytokine profiles, tumor-infiltrating immune cell composition, and functional orientation (immune activation and suppression), leading to significant differences in clinical prognosis. This study provided a conceptual framework for understanding the TIME of TNBC, which has potential clinical significance for designing novel immunotherapy and appropriate combination strategies.

## 2 Materials and Methods

### 2.1 Source of Expression Profile Data (METABRIC and GEO)

The cBioPortal database is an open and freely available resource for storing and exploring multiple cancer genomics datasets. We downloaded the METABRIC BC data from the cBioPortal database, including the collected clinical information, gene expression profile, and mutation data (https://www.cbioportal.org/study/summary?id=brca_metabric) (299 TNBC samples, S1_metabric_brca_cli.txt). GEO data were downloaded from the Gene Expression Omnibus (GEO), and the GSE58812(107 samples) dataset with survival time was selected (S2_GEO_TNBC_Clinical.txt), and samples without survival status were removed.

### 2.2 Source of Immune-Related Genes

The following categories of genes were collected as immune-related genes for subsequent analysis by literature mining (Breuer et al., 2013):

i) immune cell-specific genes derived from unicellular RNA-seq data;
ii) genes encoding costimulatory and costimulatory molecules;
iii) genes encoding cytokines and cytokine receptors;
iv) genes involved in antigen processing and presentation, and other immune-related genes.

Finally, a total of 2,006 immune-related genes were collected (S3_Immune_related_genes.v2.txt).

### 2.3 Identification of Immune Subtypes and Gene Modules and Assessment of Relevant Characteristics

The “ConsensusClusterPlus” package was used to construct the consistency matrix and clustering classification of the TNBC samples (Wilkerson and Hayes, 2010). The immune subtypes of the samples were obtained by screening the expression data of the immune-related genes. We performed 500 bootstraps using the PAM algorithm and adopted “Canberra” as a measure of distance. Each bootstrap process involved 80% of patients from the training set. The clustering number was set as 2–10. Then, we calculated the consistency matrix and cumulative consistency distribution function to determine the best classification. At the same time, the immune genes were grouped by consistent clustering with identical settings and parameters to obtain immune gene modules.

In the METABRIC dataset (training set), the prognostic value of IS was evaluated by the log-rank test and univariate and multivariate Cox regression with clinical information as the covariate and overall survival (OS) as the endpoint. Then, in the GSE58812 dataset (validation set), the association of immune subtypes with various immune-related molecular and cellular characteristics was evaluated using ANOVA.

### 2.4 Functional and Single-Sample Gene Set Enrichment Analysis

First, we annotated the biological function of the immune gene modules. Then, we performed DAVID (Version 6.8) to annotate the biological process of genes from each module in the Gene Ontology database, taking immune-related genes collected as the background. The association of immune subtypes with immune-related molecular and cellular characteristics was evaluated using an ANOVA algorithm (Thorsson et al., 2018).

ssGSEA defines an enrichment score, representing the absolute degree of enrichment of the gene set in each sample in a given data set. Sort and normalize the gene expression values of a given sample, sort the genes by their absolute expression levels, and calculate their empirical cumulative distribution differences. In order to compare the distribution of immune cell components in different immune subtypes, we obtained 28 kinds of immunity. The marker genes of the cells are scored for each type of immune cell to determine the score of 28 types of immune cells in each patient.

### 2.5 Prediction of Immune and Chemical Reactions

The RNA-Seq expression profile of METABRIC-TNBC samples combined with subclass mapping was used to predict the clinical response of TNBC immune subtypes to immunotherapy drugs. Using subclass mapping, we compared the similarity of the immunotherapy patients between three immune subtypes and the GSE91061 dataset, with a low *p*-value corresponding to a high similarity. Then, we predicted the chemotherapy response in each sample based on the Genomics of Drug Sensitivity in Cancer (GDSC, https://www.cancerrxgene.org/). Four chemotherapeutic agents commonly used in TNBC were selected: cisplatin, paclitaxel, gemcitabine, and gefitinib. The R package “pRRophetic” was used to predict the IC_50_ of the four drugs.

### 2.6 Immune Landscape

Considering the dynamic characteristics of the immune system, we used a graph-based learning method for dimension reduction analysis to reveal the immune system’s internal structure and visualize the distribution of individual patients. This analysis allows high-dimensional gene expression data to be projected into the tree structure of low-dimensional space by retaining local geometric information. This method simulated cancer progression and defined the developmental trajectory of single-cell gene expression data (Sun et al., 2014; Trapnell et al., 2014). In this study, we extended the analysis to the immune gene expression profile. The immune landscape reflected the relationship between patients in a nonlinear manifold, which may complement the discrete immune subtypes defined in linear Euclidean space.

### 2.7 WGCNA Analysis

The R package WGCNA was used to identify the co-expression modules of the immune genes, The research showed that the co-expression network was scale-free. In other words, the logarithm (log(k)) of the occurrence of nodes with a connectivity degree of k was negatively correlated with the logarithm (log(P(k))) of the occurrence probability of this node (correlation coefficient >0.85). Next, the representation matrix was transformed into an adjacency matrix, then transformed into a topological matrix. We utilized the average-linkage hierarchical clustering method to cluster the target genes based on the topological overlap matrix (TOM). Moreover, the standard for hybrid dynamic clipping trees was required, and the minimum number of genes in each network module was set as 30. Then, we calculated the eigengenes of each module successively once the gene modules were identified. Cluster analysis was performed to combine the modules close to each other into new modules (height = 0.25, deep split = 2, min module size = 30).

### 2.8 Statistical Analysis

All statistical analyses were conducted using R (Version 3.6.3). The χ2 test or Fisher exact test was used for categorical variables. A *p*-value <0.05 was considered to be statistically significant.

## 3 Results

### 3.1 Molecular Typing Based on Immune-Related Genes

**FIGURE 1:**
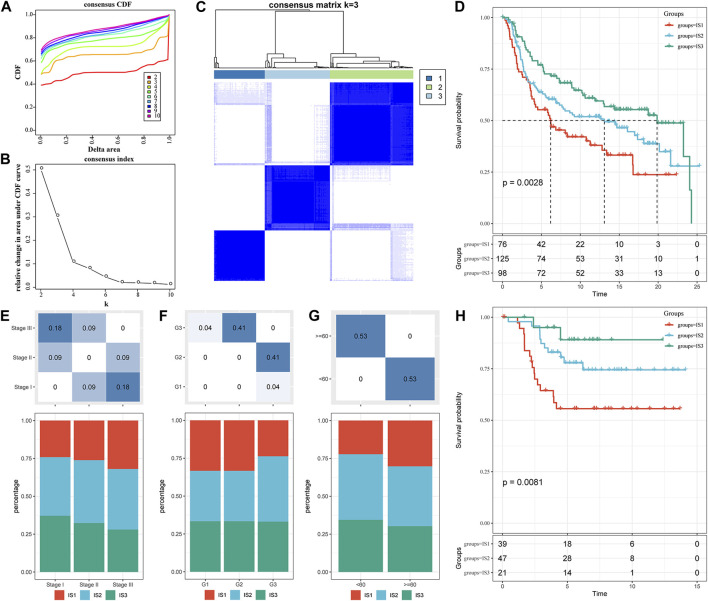
The immune subtypes in the METABRIC-TNBC cohort. **(A)** CDF curves of the METABRIC-TNBC cohort samples. **(B)** CDF Delta area curve of the METABRIC-TNBC cohort sample. The Delta area curve of the consensus clustering indicates the relative change in area under the cumulative distribution function (CDF) curve for each category number k compared with k−1. The horizontal axis represents the category number k, and the vertical axis represents the relative change in area under the CDF curve. **(C)** Consensus matrix, the heat map of sample clustering when k = 3. **(D)** KM curve of the prognosis among the three immune subtypes. **(E)** Proportion of immune molecular subtypes in different stages of the METABRIC-TNBC cohort. **(F)** Proportion of immune molecular subtypes in different grades of the METABRIC-TNBC cohort. **(G)** Proportion of immune molecular subtypes in different ages of the METABRIC-TNBC cohort. **(H)** Prognostic differences among the three immune molecular subtypes in the GSE58812 cohort. The lower part is the proportion, and the upper part is the distribution difference between the two parts, with a statistical significance of *p* = −log10.

A total of 1,702 immune-related genes in the METABRIC-TNBC microarray were obtained. The 299 TNBC samples were clustered by “ConsensusClusterPlus.” Furthermore, we obtained three immune subtypes (Figure 1C; S4_METABRIC_TNBC_subtype.txt) in terms of cumulative distribution function (CDF) (Figures 1A,B). Further analysis of prognostic characteristics revealed a better prognosis for IS3 and a worse prognosis for IS1 (Figure 1D, *p* = 0.0028). We observed no significant differences in age, stage, or grade among the three immune subtypes (Figures 1E–G). In addition, the analysis of the GSE58812 dataset suggested a conclusion consistent with the METABRIC dataset. IS3 had the best prognosis among the three immune subtypes, while IS1 had the worst prognosis, showing significant differences (Figure 1H, *p* = 0.0081).

### 3.2 Evaluation of Immune Subtypes and Related Clinical Characteristics

#### 3.2.1 Association of Immune Subtypes With TMB and Common Gene Mutations

**FIGURE 2:**
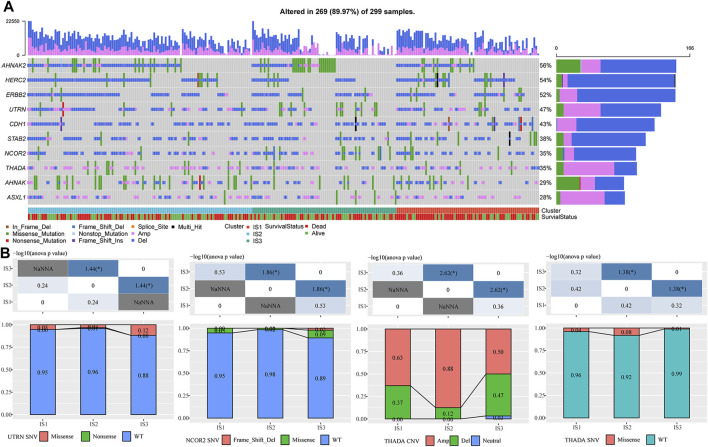
**(A)** Mutation characteristics of significantly mutated genes in each IS. **(B)** Difference analysis of UTRN, NCOR2, and ThADA genes in immune subtypes. The chi-square test determined the *p*-value. *p* < 0.05 is represented by *, while *p* < 0.01 is represented by **.

We downloaded a mutation dataset and a copy number variation dataset from the METABRIC-TNBC sample (targeted sequencing of 173 genes), and through conditional screening (mutation frequency >3; significantly high-frequency mutation genes (*p* < 0.05)), we finally obtained ten genes (metabric.subtype.mut.sig.gene.txt; Figure 2A). The results suggested that NCOR2 and UTRN had significant differences between IS2 and IS3 in the mutant samples. However, THADA showed significant differences between IS2 and IS3 in both the amplified and deleted samples, with more amplification occurring in IS2 than in IS3 (Figure 2B).

#### 3.2.2 Expression of Classic Markers of Chemotherapy-Induced Immune Responses and Immune Checkpoint Genes in the Immune Subtypes

**FIGURE 3:**
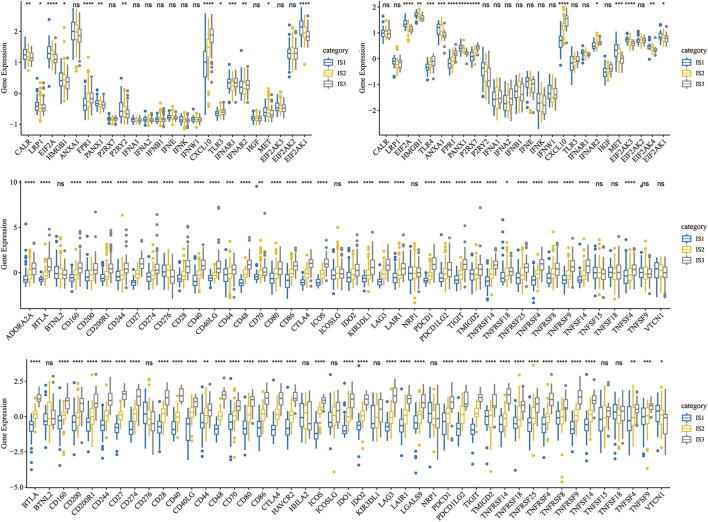
**(A)** Differential expression distribution of classic markers induced by chemotherapy in the METABRIC-TNBC cohort. **(B)** Differential expression distribution of classic markers induced by chemotherapy in the GSE58812 cohort. **(C)** Differential expression distribution of immune checkpoint genes in the METABRIC-TNBC cohort. **(D)** Differential expression distribution of immune checkpoint genes in the GSE58812 cohort. ANOVA tested significant differences. * represents *p* < 0.05, ** represents *p* < 0.01, *** represents *p* < 0.001, and **** represents *p* < 0.0001.

To observe the expression distribution of classical markers in the chemotherapy-induced immune response among the three immune subtypes, we calculated gene differences in the METABRIC-TNBC cohort and GSE58812 dataset. In the METABRIC-TNBC cohort, 13/24 genes verified significant differences in each subtype (Figure 3A). In the GSE58812 cohort, 15/26 genes showed significant differences in each subtype (Figure 3B). These results indicated differences in chemotherapy-induced immune response markers among the different immune subtypes, which may contribute to the different clinical progression of tumors.

Simultaneously, we obtained 47 immune checkpoint-related genes (Danilova et al., 2019) and, significant differences were found in 40 (85.1%) genes in the METABRIC-TNBC and 39 (83.0%) genes in the GSE58812 (Figures 3C,D).

#### 3.2.3 Immune Characteristics in Different Immune Subtypes

**FIGURE 4:**
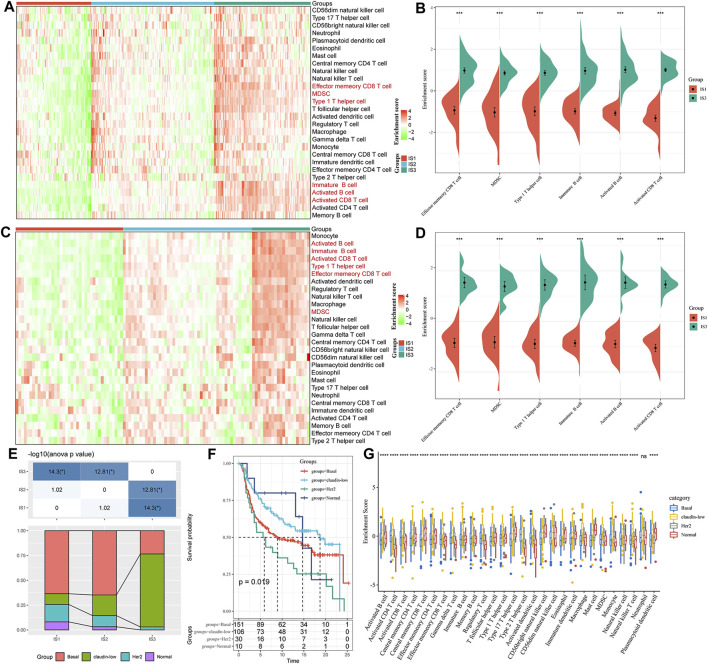
**(A)** Enrichment score difference of 28 immune cells among immune subtypes in the METABRIC-TNBC cohort. **(B)** There are several immune cell enrichment scores with significant differences between good and poor prognosis subtypes in the METABRIC-TNBC cohort. **(C)** Enrichment score difference of 28 immune cells among immune subtypes in the GSE58812 cohort. **(D)** There are several immune cell enrichment scores with significant differences between good and poor prognosis subtypes in the GSE58812 cohort. **(E)** Intersection of three immune subtypes with PAM50 molecular subtypes. **(F)** Survival analysis of the PAM50 analysis subtype. **(G)** Distribution of 28 immune cell enrichment scores in the three immune subtypes.

To compare the distribution of immune cell components in the different immune subtypes, we obtained 28 immune cell marker genes (Charoentong et al., 2017). Based on the ssGSEA method, we calculated 28 immune scores of corresponding patients in the Metabric-TNBC and GSE58812 cohorts (Figure 4A). The numbers of effector memory CD8 T cells, MDSCs, type 1 T helper cells, immature B cells, activated B cells, and activated CD8 T cells were significantly lower in IS1 than in IS3 (Figure 4B). Similar findings were also observed in the GSE58812 cohort, suggesting that the poor prognosis of TNBC may be related to cellular inhibition (Figures 4C,D).

At the same time, we explored the relationships among the three immune subtypes and the PAM50 molecular classification. We found that the proportion of Basal subtype in IS1 and IS2 was significantly higher than that of IS3; the proportion of claudin-low type in IS3 was significantly higher than that of IS1 and IS2 (Basal_BC-the worst prognosis, and claudin-low_BC-better prognosis. Figure 4E); We analyzed the prognostic differences of the PAM50 molecular subtypes of TNBC and indicated that there are significant differences in the prognosis of the PAM50 molecular subtypes (Figure 4F); In addition, the immune cell scores are significantly different in PAM50 subtypes (Figure 4G), indicating that immune cells (activated CD8 T cells, activated CD4 T cells, type 1 T helper cells, immature B cells and activated B cells etc.) have a higher proportion of claudin subtype with better prognosis.

### 3.3 Difference Analysis of Immune Subtypes in Response to Immunotherapy/Chemotherapy

**FIGURE 5:**
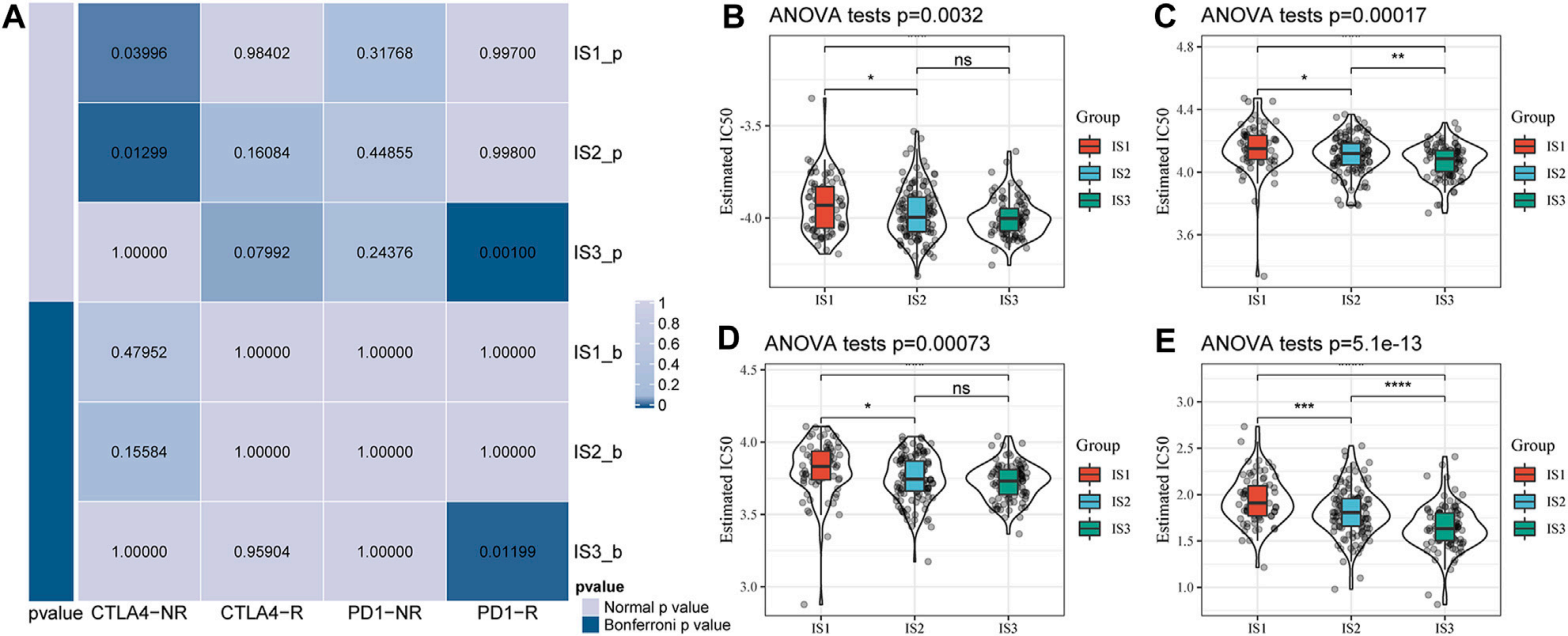
**(A)** Submap analysis showed that IS3 could be more sensitive to the programmed cell death protein one inhibitor (Bonferroni-corrected *p* < 0.05). Box plots of the estimated IC_50_ for paclitaxel **(B)**, veliparib **(C)**, olaparib **(D)**, and talazoparib **(E)**.

We further analyzed the differences among the different immune subtypes for the responses to immunotherapy and chemotherapy. The results suggested that IS3 was more sensitive to programmed cell death-1 (PD-1) inhibitors than the other two subtypes (Figure 5A). We also analyzed the responses of different subtypes to several traditional chemotherapy drugs, including paclitaxel, veliparib, olaparib, and talazoparib, and found that IS3 was more sensitive to these drugs than the other subtypes (Figures 5B–E).

### 3.4 The Immune Landscape of Triple-Negative Breast Cancer

**FIGURE 6:**
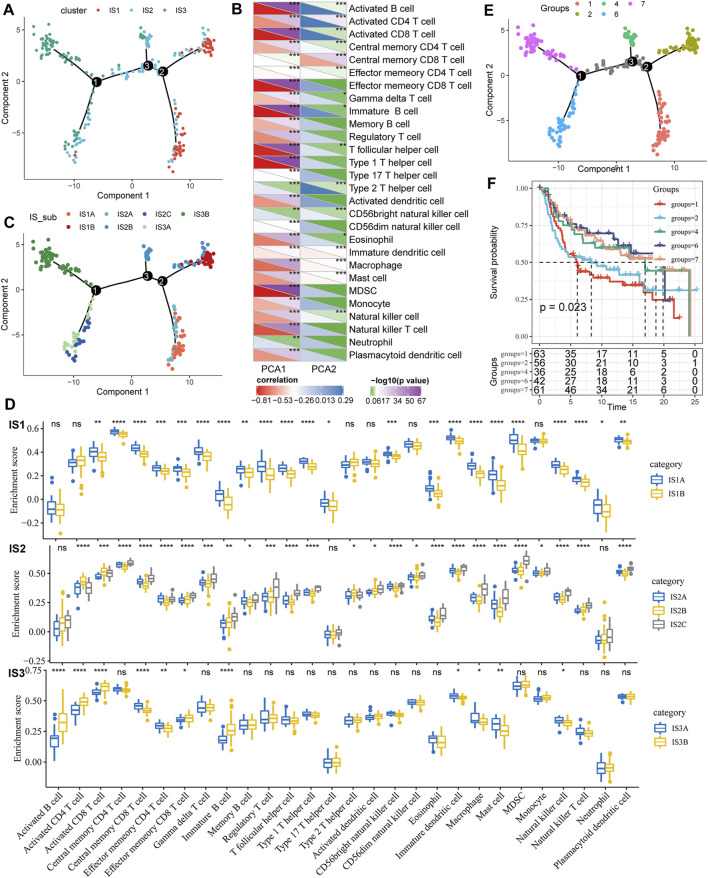
**(A)** Immune landscape in TNBC. Each dot represents a sample, and each color represents a molecular subtype. The horizontal axis represents the first principal component, while the vertical axis represents the second principal component. **(B)** Heat map of the correlation between two principal components and 28 immune cell types. **(C)** Immune landscape and molecular subgroups of three immune subtypes in TNBC. **(D)** Immune landscapes and samples from two different locations in TNBC. **(E)** Immune landscape in TNBC. **(F)** Prognostic differences among samples at different locations in the immune landscape for TNBC.

To visualize and reveal the potential structure of the individual distributions, we applied dimensionality reduction based on graph learning to the expression profile of the immune-related genes. This analysis placed each patient into a graph with a sparse tree structure, defined as the immune landscape of TNBC. The position of the patients represented the general characteristics of the tumor microenvironment in the corresponding subtype (Figure 6A). The horizontal coordinates were highly correlated with a variety of immune cells. The horizontal coordinates had the highest correlation with type 1 T helper cells, image B cells, MDSCs, effector memory CD8 T cells, activated CD8 T cells, and activated B cells (Figure 6B, |*R*| > 0.8). However, vertical coordinates showed the highest correlation with central memory CD8 T cells, activated CD4 T cells, and immature dendritic cells (DCs). Interestingly, IS1 was vertically distributed at opposite ends of the immune landscape, suggesting significant intraclass heterogeneity in each subtype. According to the position of IS3 in the immune landscape, it could be further divided into two subtypes (Figure 6C), which exhibited specific immune expression patterns (Figure 6D). In addition, different locations of the immune landscape also indicated different prognostic characteristics (Figures 6E,F). In summary, the analysis of the immune landscape provided further complementary results for the immune subtypes we mentioned earlier.

### 3.5 Construction of Immune Gene Modules and Functional Analysis

#### 3.5.1 Identification of Immune Gene Co-Expression Modules

**FIGURE 7:**
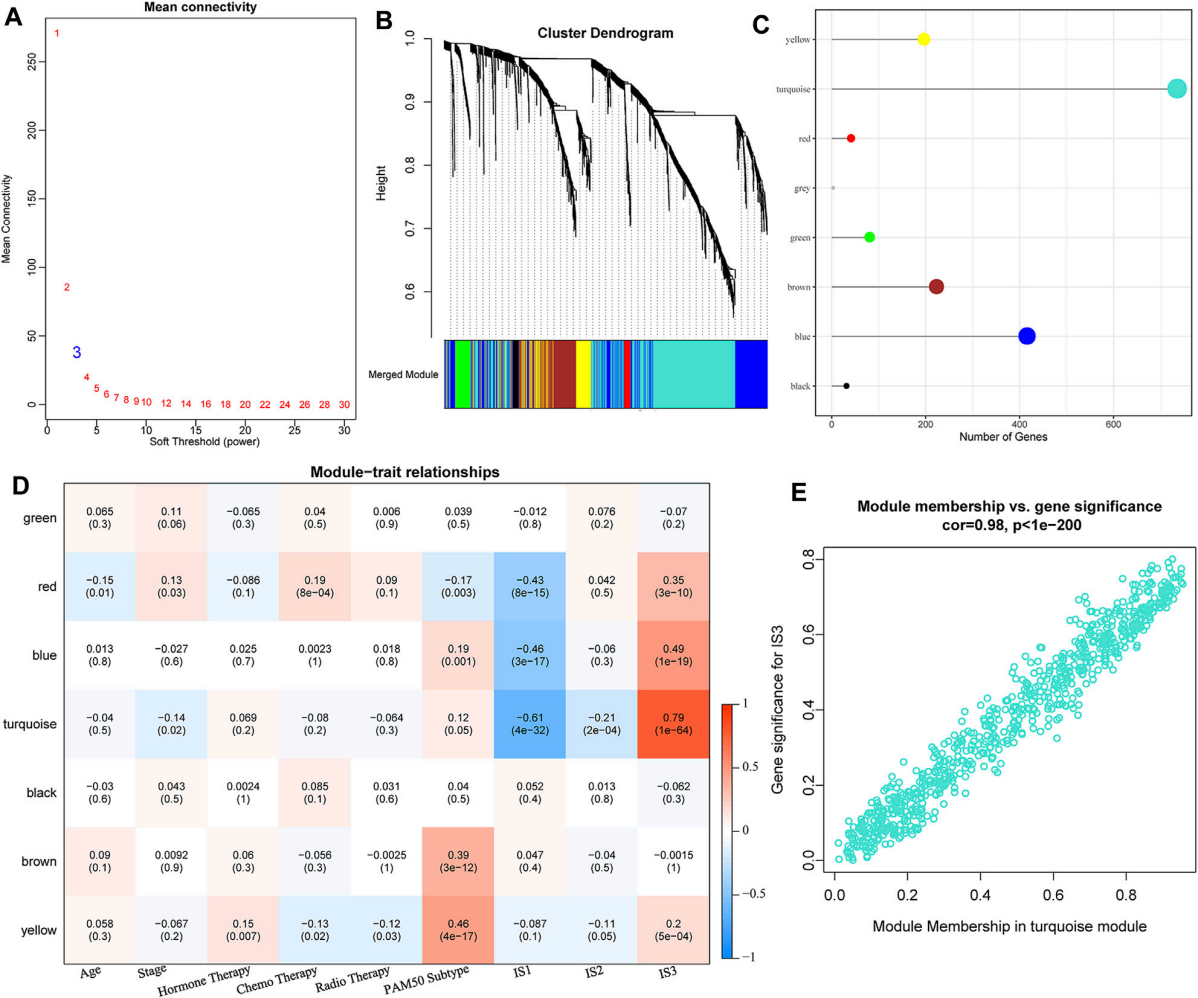
**(A)** Analysis of network topology for various soft-thresholding powers. **(B)** Gene dendrogram and module colors. **(C)** Distribution of gene numbers in seven modules. **(D)** Heatmap of the correlations between seven modules and three subtypes. **(E)** Scatter diagram of module membership vs. gene significance for IS3 in the turquoise module.

We clustered samples-expression (soft threshold = 3) and chose *β* = 10 to ensure a scale-free network (Figure 7A) using WGCNA. Finally, a total of seven modules were obtained (Figures 7B,C). We further analyzed the correlation of each module with clinical characters (Figure 7D). Among these, the highest correlation was detected between the turquoise module and IS3. The correlation between gene significance (GS) and module membership (MM) of genes within the turquoise module was also analyzed, indicating a high positive correlation between each other (S5_METABRIC_TNBC_Module_gene_cor.txt, Figure 7E).

#### 3.5.2 Functional and Prognostic Analysis of Immune Gene Co-expression Modules

**FIGURE 8:**
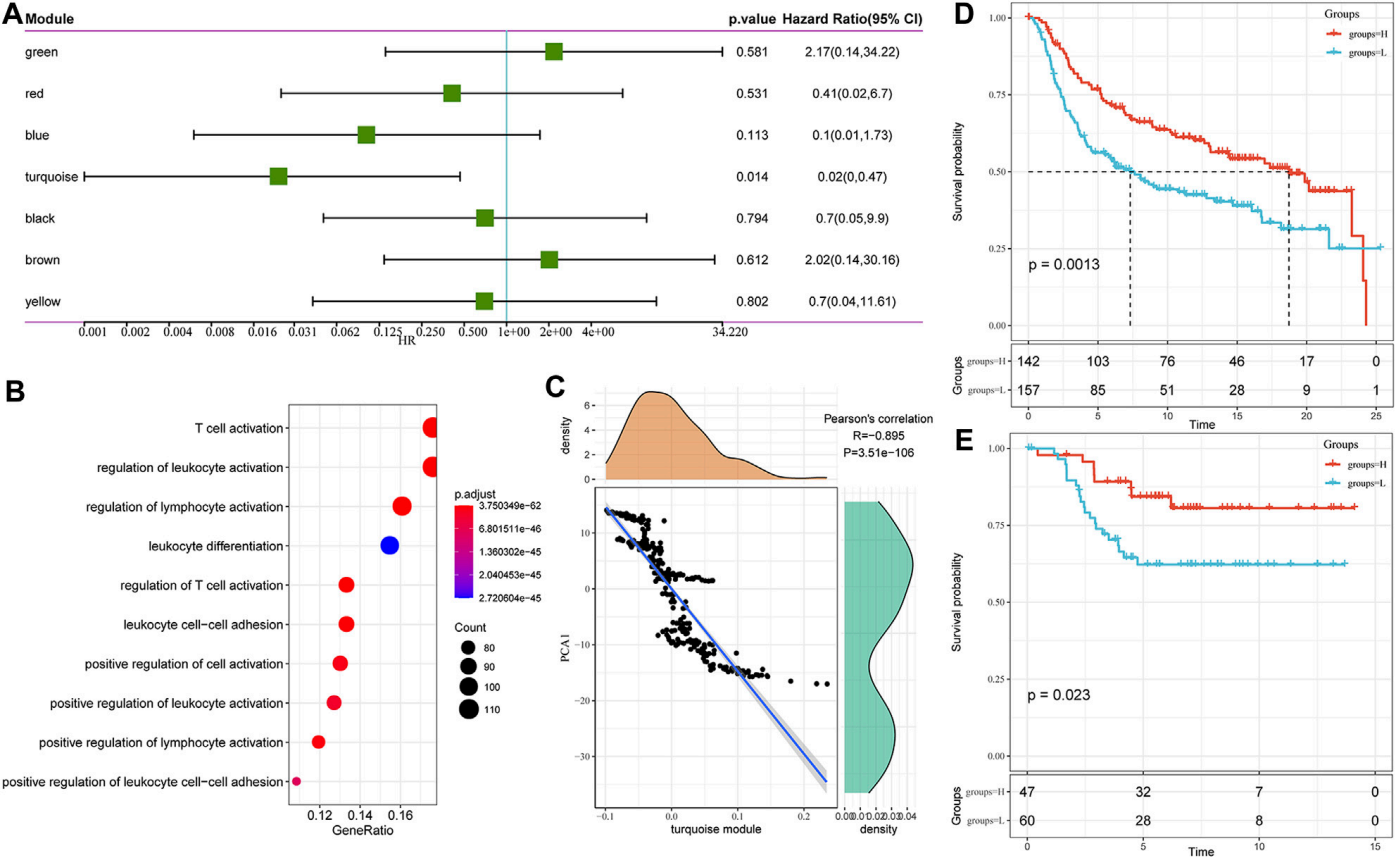
**(A)** Univariate analysis results of gene module eigengenes. **(B)** Gene enrichment analysis of the turquoise module. **(C)** Correlation between the turquoise module eigengene and the first principal component in the immune landscape. **(D)** KM survival curve distribution of patients grouped in the METABRIC-TNBC cohort according to the expression of model characteristic genes screened from the turquoise module. **(E)** KM survival curve distribution of patients grouped in the GSE58812 cohort according to the expression of model characteristic genes screened from the turquoise module.

We found that some gene modules were significantly associated with the prognosis of TNBC by identifying all immune-related gene modules (Figure 8A). For example, a low score for the turquoise module predicted a poor outcome. Functional enrichment analysis illustrated that the turquoise module was related to T cell activation, regulation of T cell activation, leukocyte differentiation, cell-cell adhesion, and other immune processes (Figure 8B). It also showed a high correlation with the first principal component in the immune landscape (Figure 8C). The gene expression profile with an eigenvector correlation coefficient greater than 0.85 from the turquoise module most related to the prognosis was extracted from the METABRIC-TNBC and GSE58812 datasets. We adopted the mean expression as the sample characteristic to classify patients and analyzed the prognostic differences between patients with high and low scores. The high score group had a significantly better prognosis in the METABRIC-TNBC dataset, and a similar phenomenon was observed in the GSE58812 dataset (Figures 8D,E). Finally, a total of five genes (correlation >0.85) to the module characteristics and the most significant prognosis in the turquoise module were selected as hub genes: RASSF5, CD8A, ICOS, IRF8, and CD247. Their low expression was revealed to be associated with a poor prognosis. In addition, we calculated the correlation between module eigengenes (ME) and gene mutations (spearman method). Based on the correlation analysis results, we screened out the top 10 genes that are most relevant to each module (S_Fig. turquoise; S_module_mutation. pdf).

## 4 Discussion

This study classified 299 TNBC samples of METABRIC-TNBC based on 1,702 immune-related cell genes. These samples can be divided into three subtypes (IS1-3), and there are significant differences in prognosis between subtypes; Immune-related genes can be divided into seven functional modules, and the distribution of immune subtypes in functional modules is different; in validation dataset (GSE58812), immune subtypes and gene modules have a high degree of reproducibility. This study provides a new perspective for understanding the tumor immune microenvironment of TNBC and may provide a reference for the design and reasonable combination of individualized immunotherapy.

First, the distribution of immune-related genes in different immune subtypes was screened and demonstrated significantly different. For example, the differential expression of NCOR2 and UTRN genes confirmed in our research has been closely related to BC’s occurrence and progression in previous studies (Li et al., 2007; van Agthoven et al., 2009). NCOR2 is nuclear receptor co-inhibitor 2, which has a background concentration in cells, plays a transcriptional inhibitory role, and participates in the regulation of multiple cell pathways such as proliferation and inflammation (Hong et al., 2001; Alrfaei et al., 2013); UTRN protein is a ubiquitous cytoskeletal protein that connects the extracellular matrix and the microfilament skeleton and maintains the integrity of the membrane structure. The destruction and disorder of microfilaments are the phenotypic characteristics of many *in vitro* cancer cell lines and a reflection of the malignant biological behavior of cancer cells (Li et al., 2007). We discuss the THADA gene (also called thyroid adenoma-associated protein) separately, located on the short arm 2p21 of chromosome 2, first discovered in thyroid tumors (Rippe et al., 2003). THADA encodes a thyroid adenoma-related protein in the thyroid, pancreas, and other tissues (Ling, 2020; Castillo-Higuera et al., 2021; Macerola et al., 2021), which is related to energy metabolism disorders. In this study, we found that the missense and amplification of this gene in the IS3 subtype were significantly lower than the other two subtypes, and its expression was consistent with a better prognostic trend. Breast and thyroid have common etiological factors and are jointly regulated by the hypothalamic-pituitary-glandular axis, and the interaction between their hormones may be one of the reasons for the coexistence of the two diseases. The differential expression of thyroid adenoma-associated proteins appears in different subtypes of TNBC patients. Can other secondary primary tumors (such as thyroid cancer) be found after follow-up, thereby increasing the detection rate of thyroid cancer? It is necessary to study this phenomenon in depth.

The TIME refers to the complex network of internal and external environments for the occurrence, growth, and metastasis of tumors, including tumor cells, immune molecules, extracellular matrix, cancer-associated fibroblasts (CAFs), tumor-associated macrophages (TAMs), and other immune-related cells (Malone et al., 2020; Qattan, 2020). On the one hand, innate and acquired immune responses mediated by immune cells and molecules can eliminate tumor cells to inhibit tumor emergence. On the other hand, the escaped tumor cell phenotype mutations form a particular immunosuppressive microenvironment, which can avoid the recognition and attack of the immune system through the loss of human histocompatibility antigenicity and the secretion of immunosuppressive molecules, which is defined as an immune escape (Liu and Cao, 2016). Multiple components of the TIME have been shown to play essential roles in tumor progression. For example, TAMs can be polarized into M2 macrophages to secrete metabolic enzymes, transcription factors, and chemokines, which play a role in promoting tumor growth (Sami et al., 2020). CAFs activated by normal fibroblasts and colony-stimulating factors secreted by DCs in the TIME were also detected to have immunosuppressive and invasion-supporting effects (Park et al., 2018; Blackley and Loi, 2019), complementary to the results of our study.

In TIME, there is a particular member to be mentioned, which is TIL. It is a mixture of anti-inflammatory and antitumor cells, 75% of T lymphocytes (Kwa and Adams, 2018). TILs are frequently detected in BC, especially in TNBC, suggesting richer immune infiltration and more significant immunogenicity. A large amount of clinical data has proven that the number of TILs is significantly positively correlated with the expected survival of TNBC patients and is an independent biomarker for patient prognosis (Park et al., 2018; Qattan, 2020). In 2019, the WHO officially listed TILs as one of the biomarkers for the clinicopathological analysis of BC (Blackley and Loi, 2019). In our study, significant differences were observed in the distribution of the number of lymphocyte members among the three immune subtypes. We found that Effector memory CD8 T cell, MDSC, Type 1 T helper cell, Immature B cell, Activated B cell, and Activated CD8 T cell is significantly lower in IS1 subtypes than IS3 subtypes. It also explains the poor prognosis of IS1. The classification of cancer subtypes according to the expression matrix is molecular typing (GEP). For example, the standard clinical GEP classification based on PAM50 (Prediction analysis of microarray 50) can divide breast cancer into different subtypes. We analyzed the relationship between the three immune subtypes and PAM50 molecular subtype; it can be seen that the subtype with poor prognosis in PAM typing has the highest proportion in IS1 or IS2 subtypes, while the subtype with better prognosis in PAM50 typing is in IS3 The highest proportion in the middle, which explains why there are prognostic differences between our subtype classifications. It can play a good role in prognostication, and it can also be used as a supplement to PAM50. Through the analysis of Figure 5G, we can see that among the subtypes with poor prognosis in PAM50, the expression of immune cells such as Activated B cell and Activated CD8 T cell is low. This phenomenon is similar to the expression of immune cells in the IS1 subtype, which is used to explain why the prognosis of Basal and Her-2 subtypes is poor.

These immune cells were mainly present in IS3 with the best tumor prognosis, we also observed that 47 immune checkpoint-related genes were significantly different in the two cohorts and were responsible for maintaining autoimmune tolerance and regulating the persistence and intensity of the immune response. Interestingly, the suppression of immune checkpoint genes has been confirmed to reverse the inhibition of the TIME on tumor immunity and accelerate the apoptosis of tumor cells, which is a potential new possibility for future treatment of TNBC (Shen et al., 2020).

Through the analysis of immune characteristics of immune subtypes, we further analyzed the immune checkpoints in different subtypes. Immune checkpoints are a class of immunosuppressive molecules. During the occurrence and development of tumors, immune checkpoints become one of the main reasons for immune tolerance. Immunotherapy has opened a new door to treating malignant tumors; therefore, many patients have achieved ideal therapeutic effects. Similarly, immunotherapy has also brought about breakthroughs in the treatment of TNBC with strong immunogenicity. At present, the most classic research direction is the PD1/PD-L1 pathway. PD1 is a group of transmembrane proteins expressed on the surface of T cells, B cells, and NK cells that promotes lymphocyte failure and inhibits tumor apoptosis by binding to the ligand PD-L1 (Francisco et al., 2009). Inhibitors targeting PD1 and PD-L1 have been treat cancer patients with some exciting but limited results. PD-L1-positive patients in Impassion130 gained an additional 7 months of overall survival (OS) after using atezolizumab, while PD-L1-negative patients showed little benefit (Simmons et al., 2020). In another group of patients with metastatic TNBC, the addition of atezolizumab did not significantly improve patient OS compared with chemotherapy alone (Gupta et al., 2020). It may be due to the different subtypes and stages of TNBC having different susceptibilities to drugs. In METABRIC-TNBC and GSE58812, there are significant differences in immune checkpoint genes between different subtypes. For example, our common CD274 (PD-L1), CTLA4, LAG3, PDCD1 (PD-1) are significantly increased in IS3, and the other two subtypes are significantly different, and most of the immune checkpoints are significantly elevated in IS3. According to the results of Immune Subtype vs. Immunotherapy/Chemotherapy Difference Analysis (Figure 6), the IS3 subtype has a lower IC50 value and higher response to Paclitaxel, Veliparib, Olaparib, and Talazoparib; compared to the other two subtypes, IS3 is more sensitive to PD-1 inhibitors. These results suggest that TNBC patients of the IS3 subtype are more sensitive to chemotherapy and immunotherapy; if patients with the IS3 subtype develop resistance during chemotherapy, immunotherapy (PD-1 inhibitors) may provide some help.

In the next step, we adopt a pseudo-chronological analysis similar to single-cell sequencing (according to changes in gene expression of different cell subgroups over time, to construct cell lineage development. A virtual time sequence refers to the transformation and evolution between cells. The order and trajectory of replacement), the dimensionality reduction method based on graph learning is used to visualize the potential distribution of patients, and the immune landscape of TNBC is constructed. Through analysis, we found significant intra-class heterogeneity in the subtypes (IS1, IS2). Coincidently, intratumor and intratumoral heterogeneity has also been noted to increase the challenge of adjuvant therapy for TNBC in several previous studies (Lee et al., 2019; Simmons et al., 2020). At present, there is still a lack of pragmatic immune markers to screen the immune predominance population.

Further studies of biomarker thresholds are necessary to separate high-risk and low-risk populations and select more personalized treatments. At the same time, we need to explore the tumor immune drivers associated with different subtypes and stages of TNBC to conduct immunotherapy more effectively. An improved preclinical model can maximize the preservation of the characteristics and clinical predictability of the human tumor-immune system for better clinical transformation. In addition, previous reports have discussed the advantages of combinations of different immunotherapies and the addition of immunotherapies to traditional treatment modalities (Gupta et al., 2020; Simmons et al., 2020). Assessing the timing, sequencing, and efficacy of combination therapies may optimize outcomes and prolong the survival of TNBC patients.

Last but not least, a more accurate prediction of the efficacy and a reduction of the adverse effects of immunotherapy is also a problem to be solved. Several biomarkers have been proposed to monitor treatment outcomes and assess the estimated survival of TNBC patients, including imaging examinations, TILs, and a pathologic complete response (PCR), while additional studies are needed to confirm these findings (Blackley and Loi, 2019; Waks and Winer, 2019; Vagia et al., 2020). In our analysis, we further divided IS3 into two subtypes (IS3A, IS3B) according to its position in the immune landscape, further refined the immune subtypes of TNBC, and provided ideas for individualized treatment in the future; The location of the drug has different prognostic characteristics, and this analysis method has specific clinical significance.

In addition, some of the seven immune-related gene modules identified were highly reproducible immune subtypes and predicted the outcomes of TNBC patients based on their scores. We also screened out the top five genes in the module most closely associated with the prognosis, including RASSF5, CD8A, ICOS, IRF8, and CD247. RASSF5 is a member of the RASSF gene family, which selectively binds and activates RAS. It has been observed to be involved in the regulation of cell differentiation and proliferation (Li et al., 2018). The RASSF5 gene is highly methylated in numerous tumors, such as neuroblastoma, oral cancer, and liver cancer, suggesting that it may be a potential tumor suppressor (Djos et al., 2012; Liu et al., 2018). Another member, IRF8, was also down-regulated in colorectal and BC tumor tissues, involved in antigen capture and response to interferon (Gatti et al., 2021). Correspondingly, high expression of IRF8 was associated with longer OS and may be related to inhibition of migration and invasion by inducing tumor cell cycle arrest and apoptosis. In addition, CD8A is mainly expressed in natural killer cells and dendritic cells and plays a cytotoxic role in tumors. An association between high expression of CD8A and better prognosis has been reported in both lung adenocarcinoma and colon cancer (Herrera et al., 2020; Ma et al., 2020). Similarly, ICOs are mainly expressed on activated cytotoxic T cells, memory T cells, and regulatory T cells, which were rarely introduced in the role of TNBC (Xiao et al., 2020). Last but not least, CD247 was thought to be involved in the recognition and presentation of tumor cells in the immune system, and its loss of function can lead to the development of immunodeficiency (Ye et al., 2019). Its functional deficits have been reported in several malignancies, including pancreatic, ovarian cancers and BC (Ishigami et al., 2002; Pappas et al., 2009).

In summary, we identified three TNBC immune subtypes and observed significant differences in several subtypes, including the prognosis, gene mutations, immune infiltration, drug sensitivity, and heterogeneity. Our research has laid a clinical foundation for the classification and additional immunotherapy of TNBC. We believe that an increasing number of TNBC patients will benefit from immunotherapy as scientific research and medical technology advance. The era of personalized immunotherapy for TNBC will finally come.

## Data Availability

The original contributions presented in the study are included in the article/Supplementary Material, further inquiries can be directed to the corresponding author.
