# Supplementary material for: Immune Classification and Immune Landscape Analysis of Triple-Negative Breast Cancer
Source: Front Genet. 2021 Nov 2;12:710534. doi: 10.3389/fgene.2021.710534 (PMC8593253; doi:10.3389/fgene.2021.710534)
Supplement: Supplementary file 1 [file DataSheet1.ZIP › S_module.pdf]

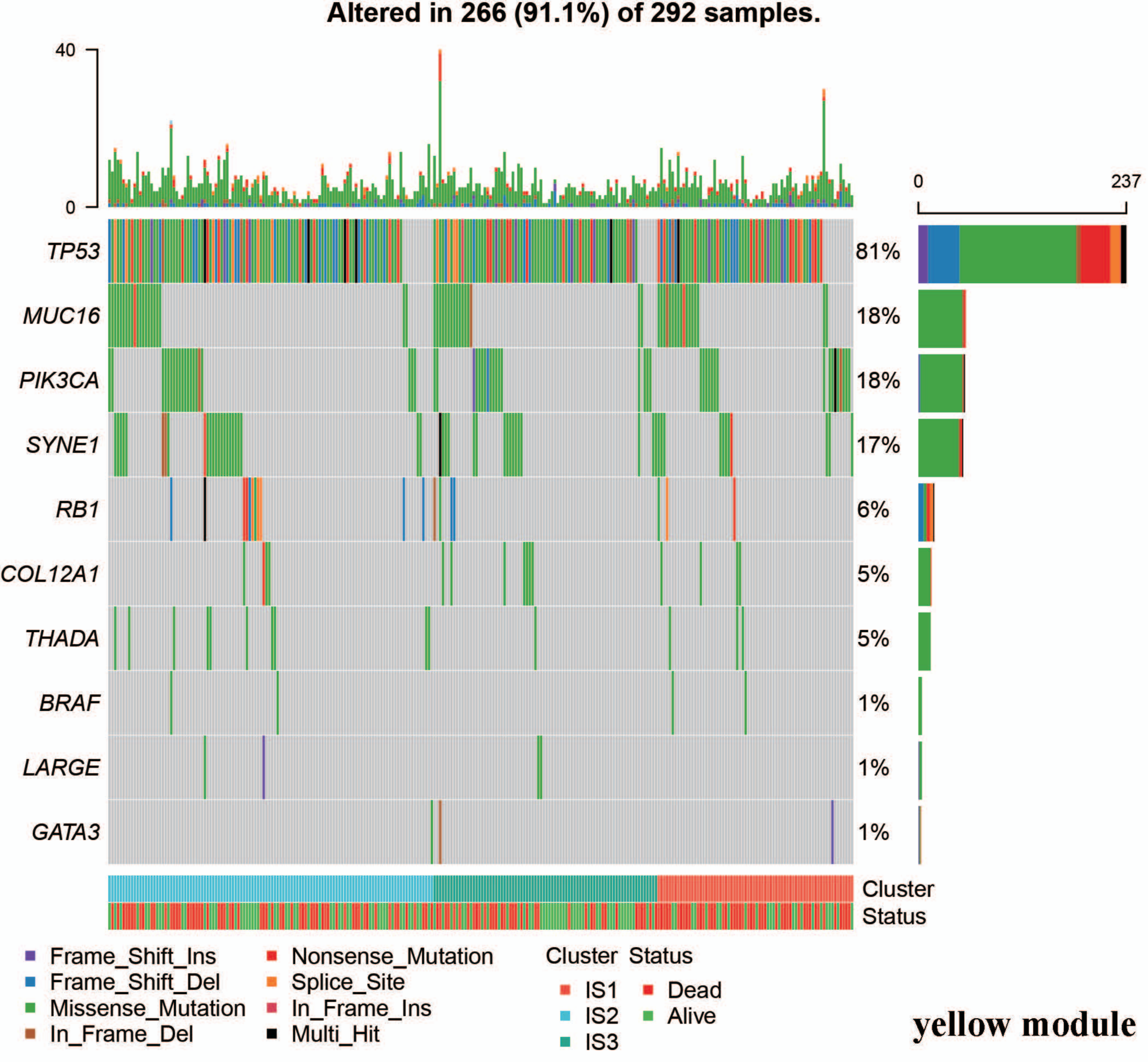

Altered in 266 (91.1%) of 292 samples.
40
0
0
237
TP53
MUC16
PIK3CA
SYNE1
RB1
COL12A1
THADA
BRAF
LARGE
GATA3
81%
18%
18%
17%
6%
5%
5%
1%
1%
1%
Cluster
Status
Frame_Shift_Ins
Frame_Shift_Del
Missense_Mutation
In_Frame_Del
Nonsense_Mutation
Splice_Site
In_Frame_Ins
Multi_Hit
Cluster
Status
IS1
IS2
IS3
Dead
Alive
yellow module

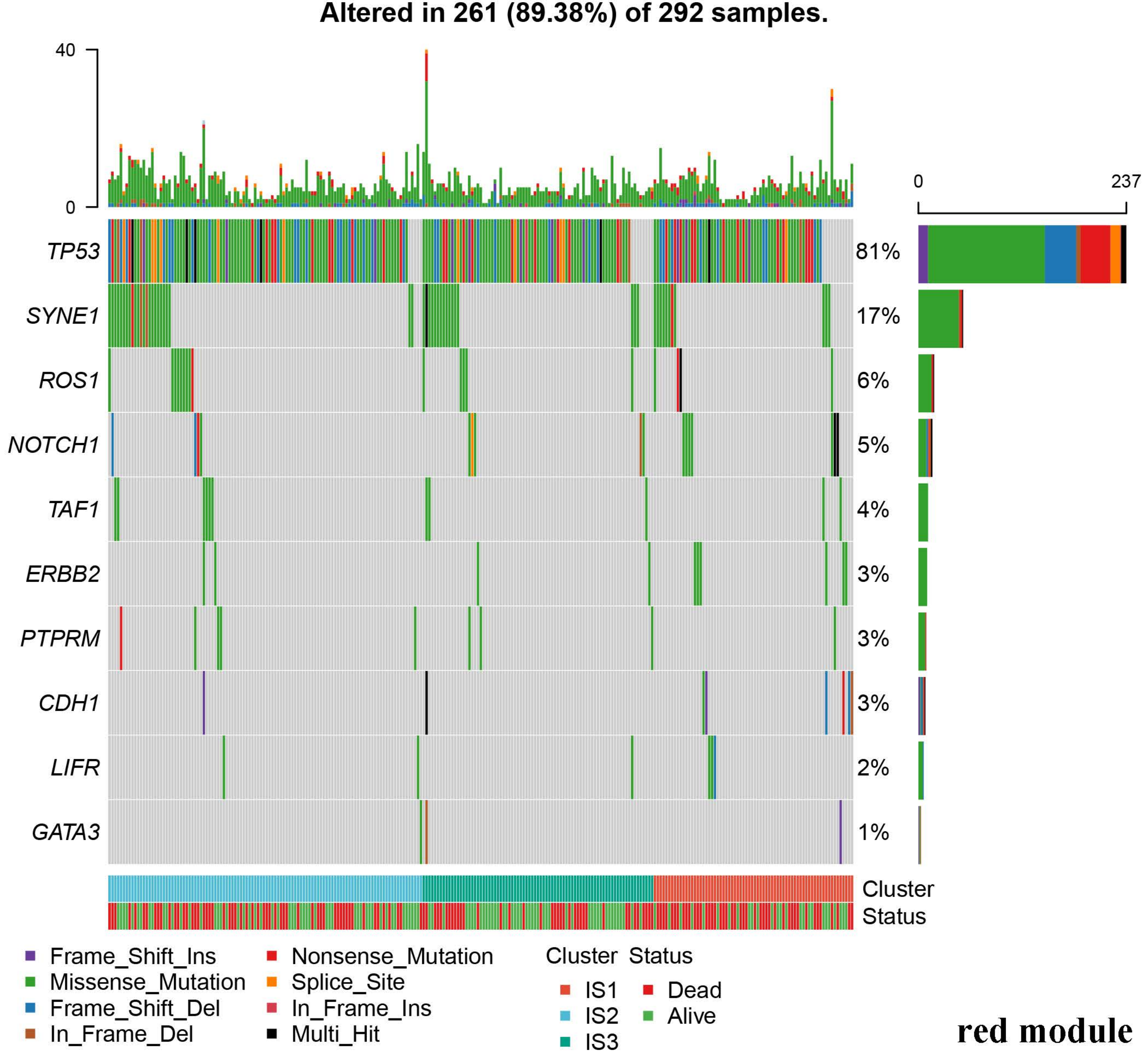

Altered in 261 (89.38%) of 292 samples.
40
0
0
237
TP53
SYNE1
ROS1
NOTCH1
TAF1
ERBB2
PTPRM
CDH1
LIFR
GATA3
81%
17%
6%
5%
4%
3%
3%
3%
2%
1%
Cluster
Status
Frame_Shift_Ins
Missense_Mutation
Frame_Shift_Del
In_Frame_Del
Nonsense_Mutation
Splice_Site
In_Frame_Ins
Multi_Hit
Cluster
Status
IS1
IS2
IS3
Dead
Alive
red module

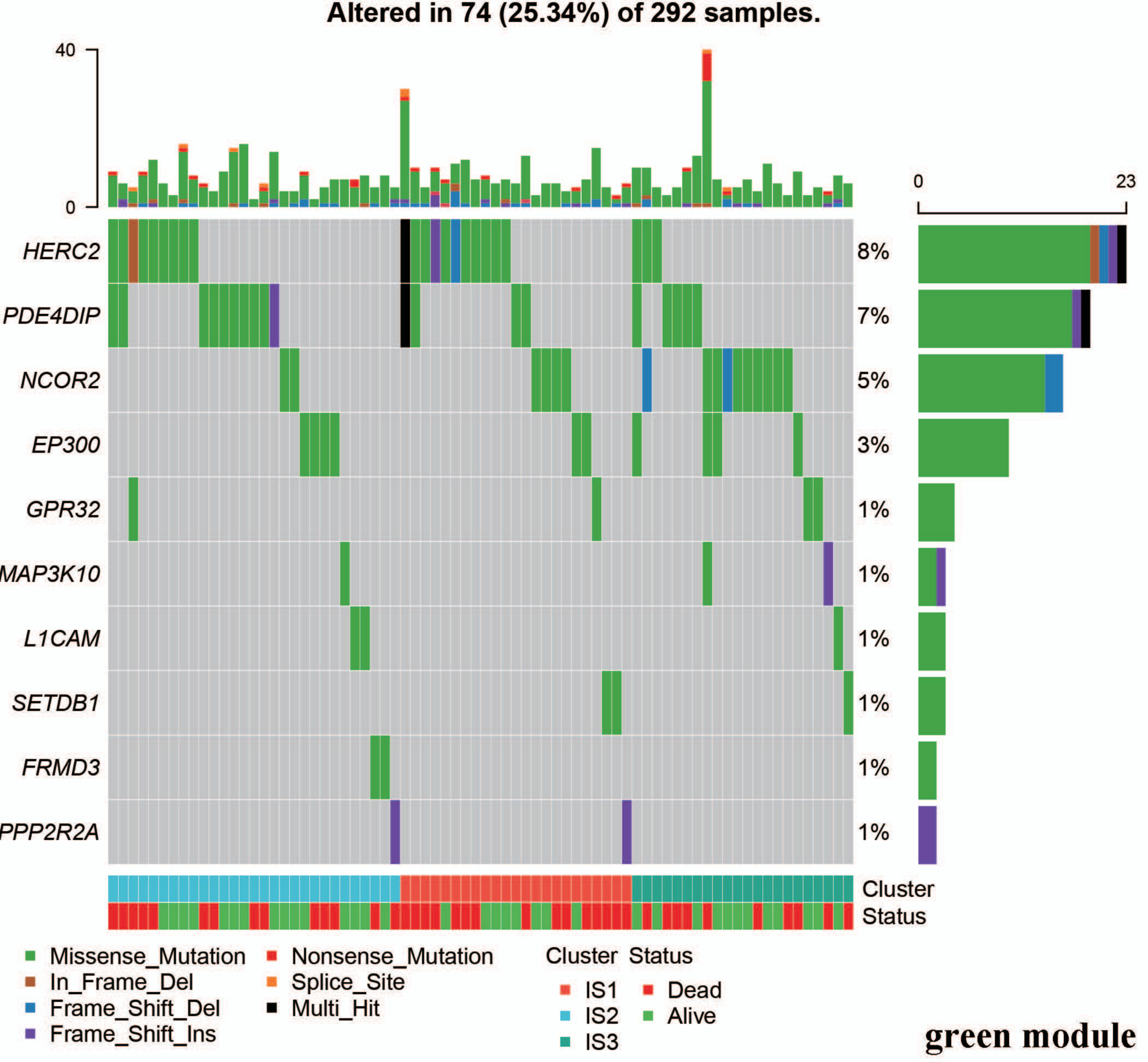

Altered in 74 (25.34%) of 292 samples.
40
0
0
23
HERC2
PDE4DIP
NCOR2
EP300
GPR32
MAP3K10
L1CAM
SETDB1
FRMD3
PPP2R2A
8%
7%
5%
3%
1%
1%
1%
1%
1%
1%
Cluster
Status
Missense_Mutation
In_Frame_Del
Frame_Shift_Del
Frame_Shift_Ins
Nonsense_Mutation
Splice_Site
Multi_Hit
Cluster
IS1
IS2
IS3
Status
Dead
Alive
green module

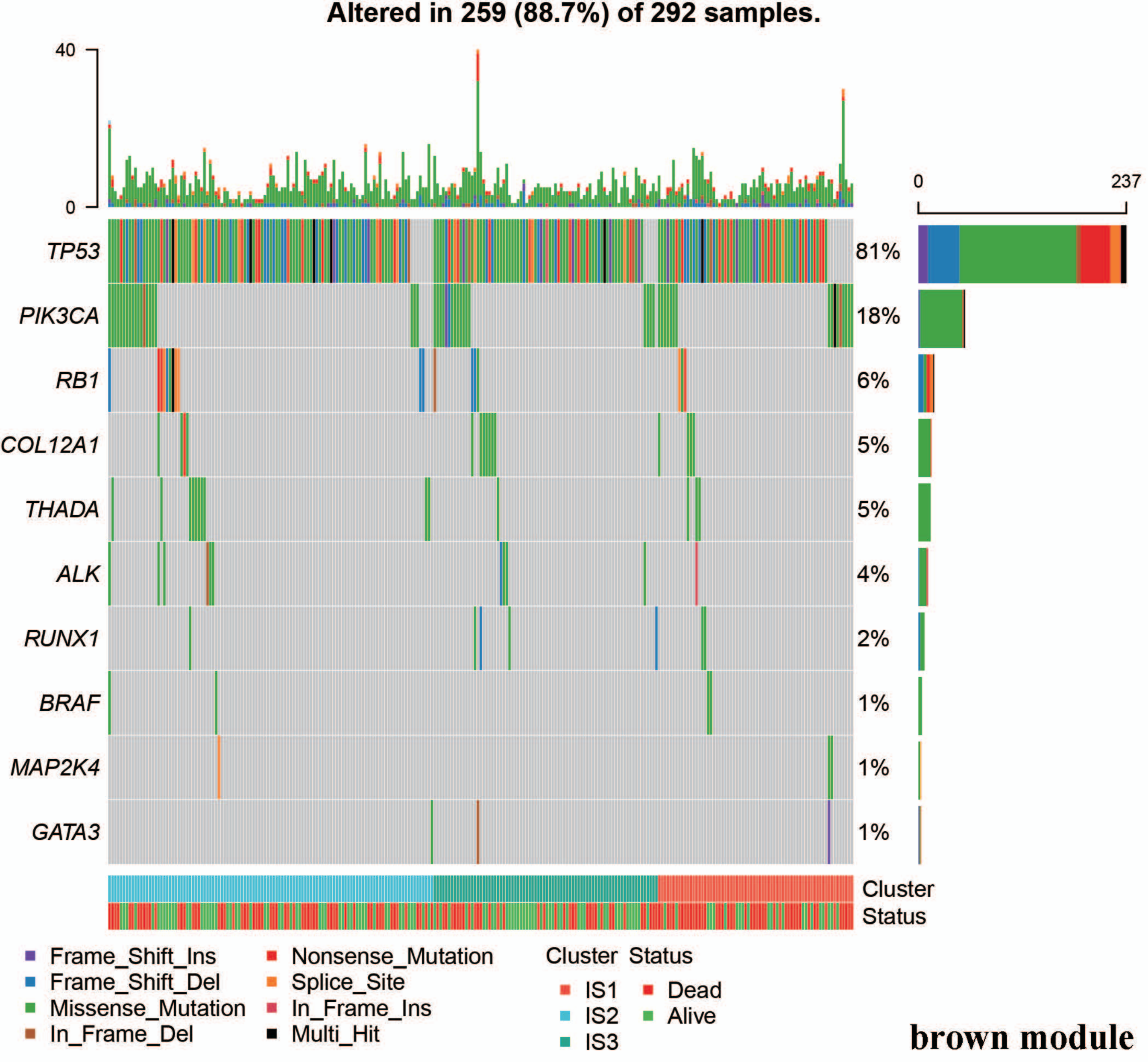

Altered in 259 (88.7%) of 292 samples.
40
0
0
237
TP53
PIK3CA
RB1
COL12A1
THADA
ALK
RUNX1
BRAF
MAP2K4
GATA3
81%
18%
6%
5%
5%
4%
2%
1%
1%
1%
Cluster
Status
Frame_Shift_Ins
Frame_Shift_Del
Missense_Mutation
In_Frame_Del
Nonsense_Mutation
Splice_Site
In_Frame_Ins
Multi_Hit
Cluster
IS1
IS2
IS3
Status
Dead
Alive
brown module

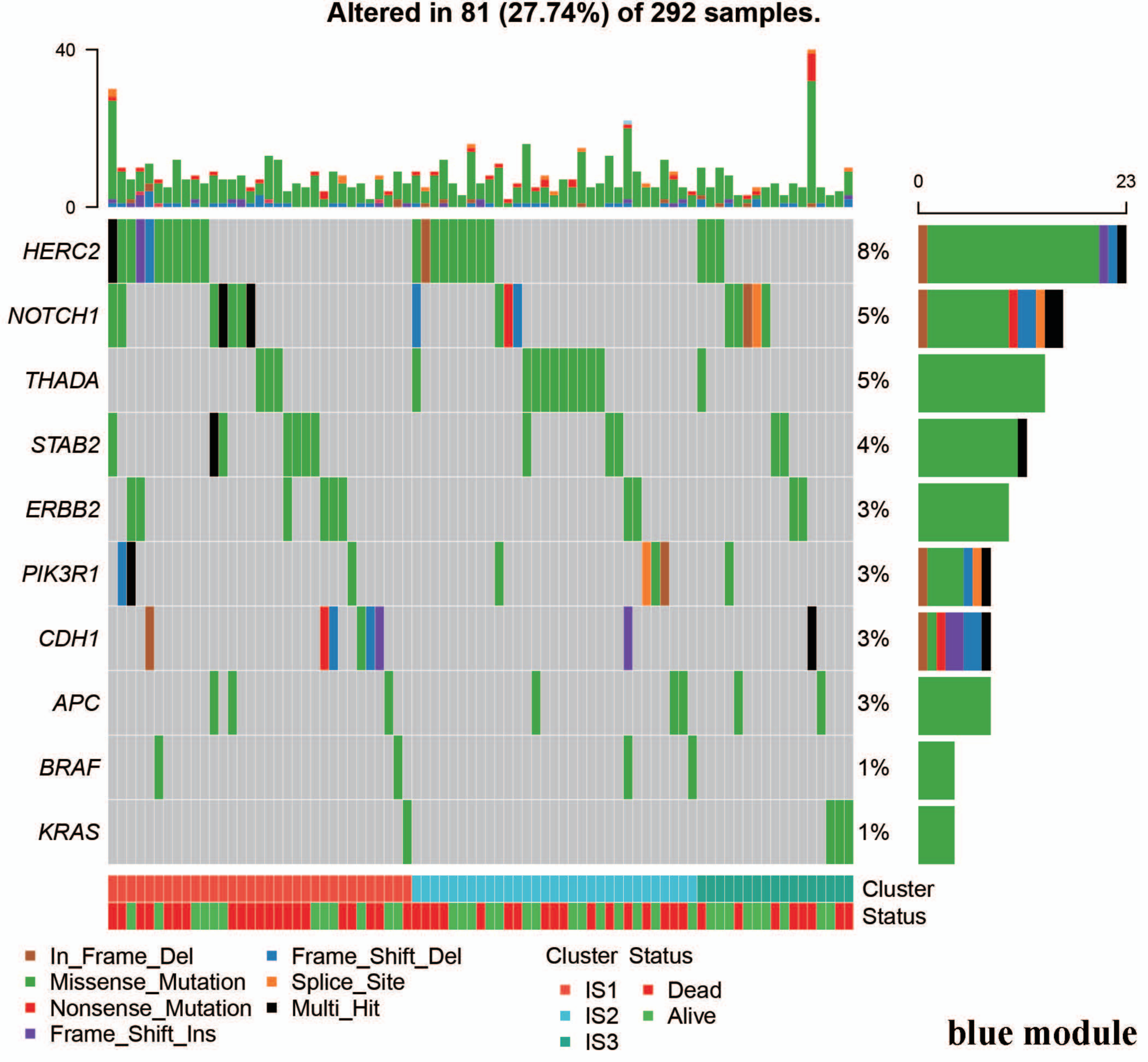
Altered in 81 (27.74%) of 292 samples.
40
0
0
23
HERC2
NOTCH1
THADA
STAB2
ERBB2
PIK3R1
CDH1
APC
BRAF
KRAS
8%
5%
5%
4%
3%
3%
3%
3%
1%
1%
Cluster
Status
In_Frame_Del
Missense_Mutation
Nonsense_Mutation
Frame_Shift_Ins
Frame_Shift_Del
Splice_Site
Multi_Hit
Cluster
Status
IS1
IS2
IS3
Dead
Alive
blue module

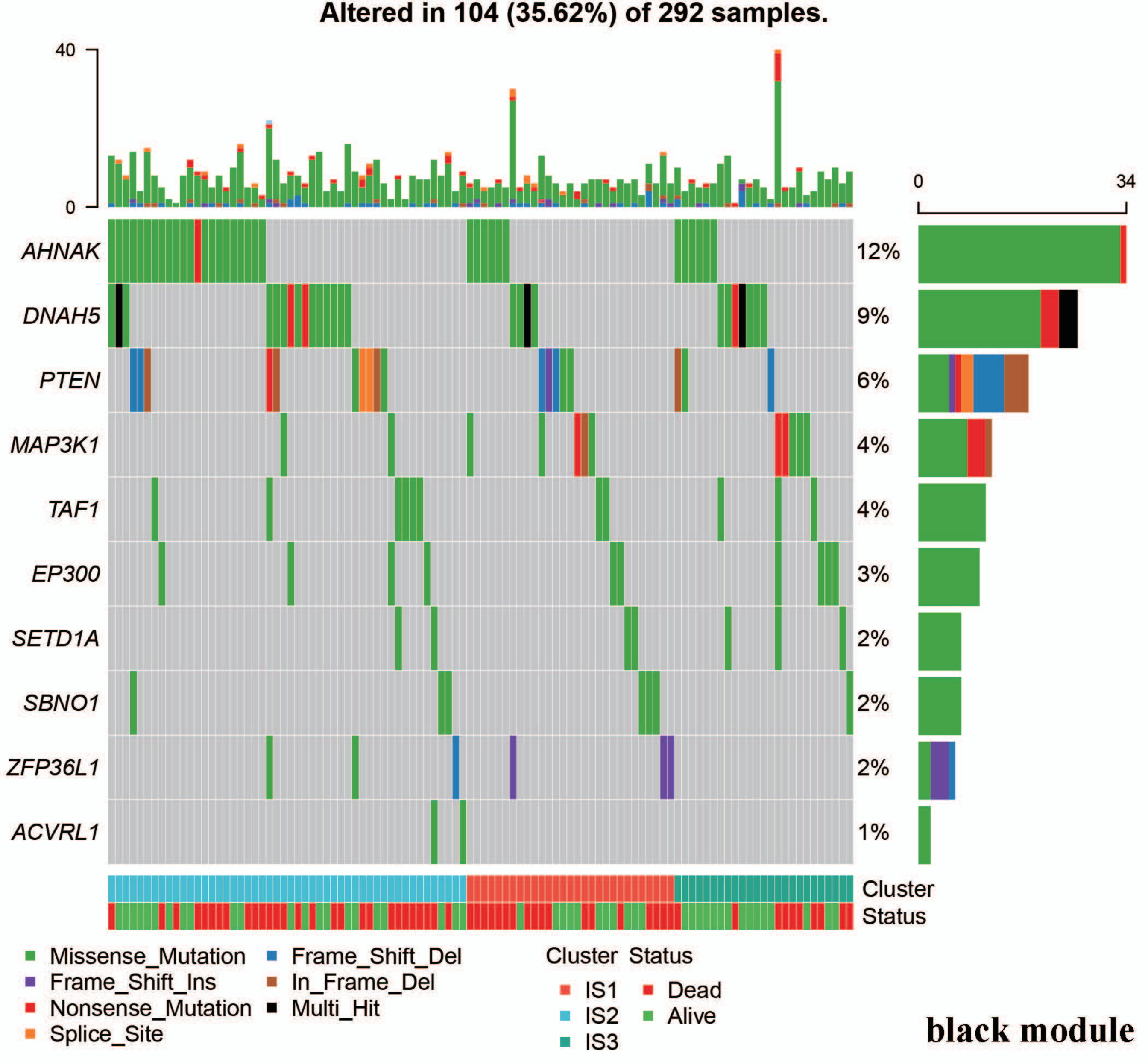

Altered in 104 (35.62%) of 292 samples.
40
0
34
AHNAK
DNAH5
PTEN
MAP3K1
TAF1
EP300
SETD1A
SBNO1
ZFP36L1
ACVRL1
12%
9%
6%
4%
4%
3%
2%
2%
2%
1%
Cluster
Status
Missense_Mutation
Frame_Shift_Ins
Nonsense_Mutation
Splice_Site
Frame_Shift_Del
In_Frame_Del
Multi_Hit
Cluster
IS1
IS2
IS3
Status
Dead
Alive
black module

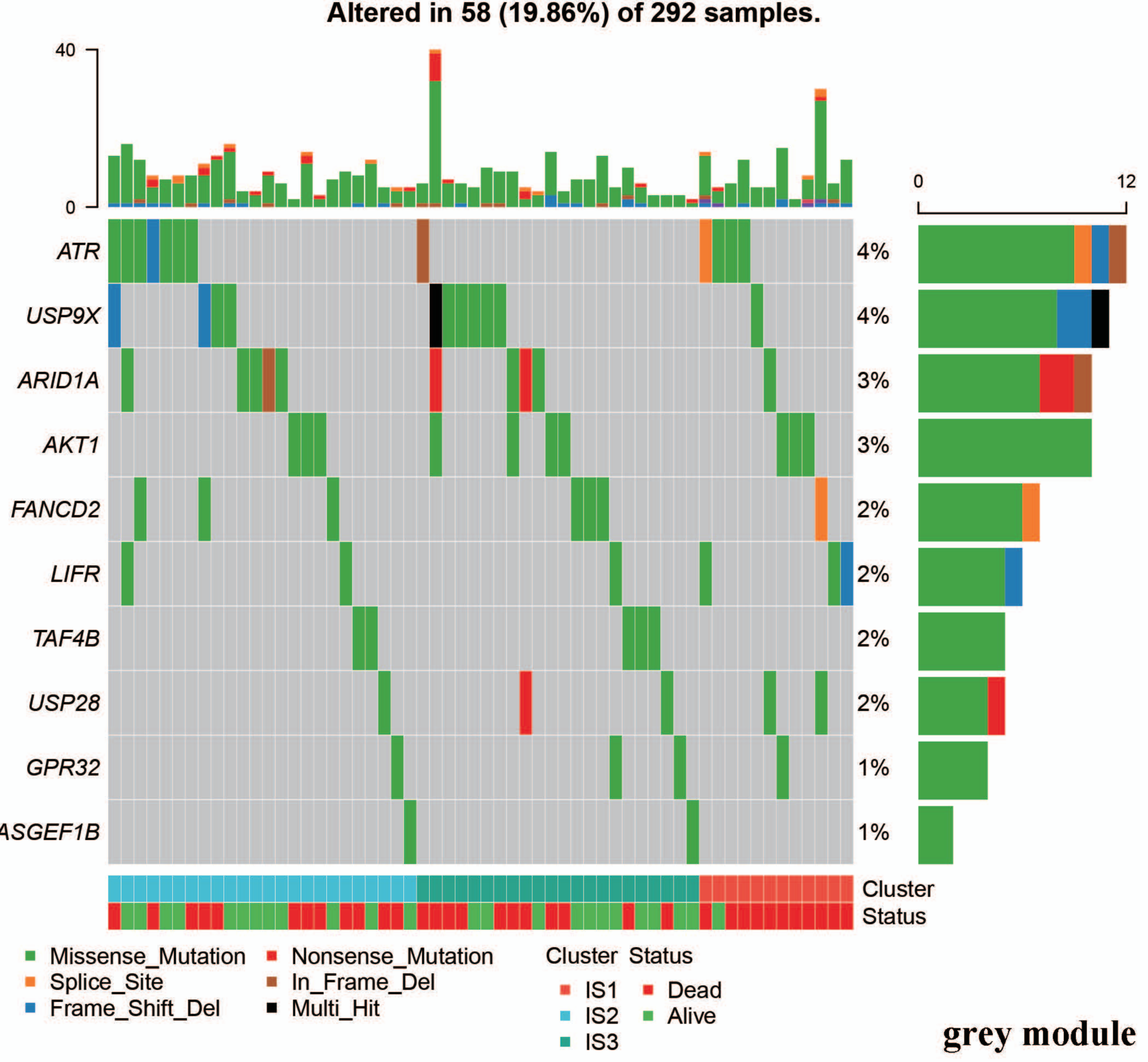

Altered in 58 (19.86%) of 292 samples.
40
0
0
12
ATR
USP9X
ARID1A
AKT1
FANCD2
LIFR
TAF4B
USP28
GPR32
ASGEF1B
4%
4%
3%
3%
2%
2%
2%
2%
1%
1%
Cluster
Status
Missense_Mutation
Splice_Site
Frame_Shift_Del
Nonsense_Mutation
In_Frame_Del
Multi_Hit
Cluster
Status
IS1
IS2
IS3
Dead
Alive
grey module
