# Supplementary figures and images for: Immune Classification and Immune Landscape Analysis of Triple-Negative Breast Cancer
Source: Front Genet. 2021 Nov 2;12:710534. doi: 10.3389/fgene.2021.710534 (PMC8593253; doi:10.3389/fgene.2021.710534)

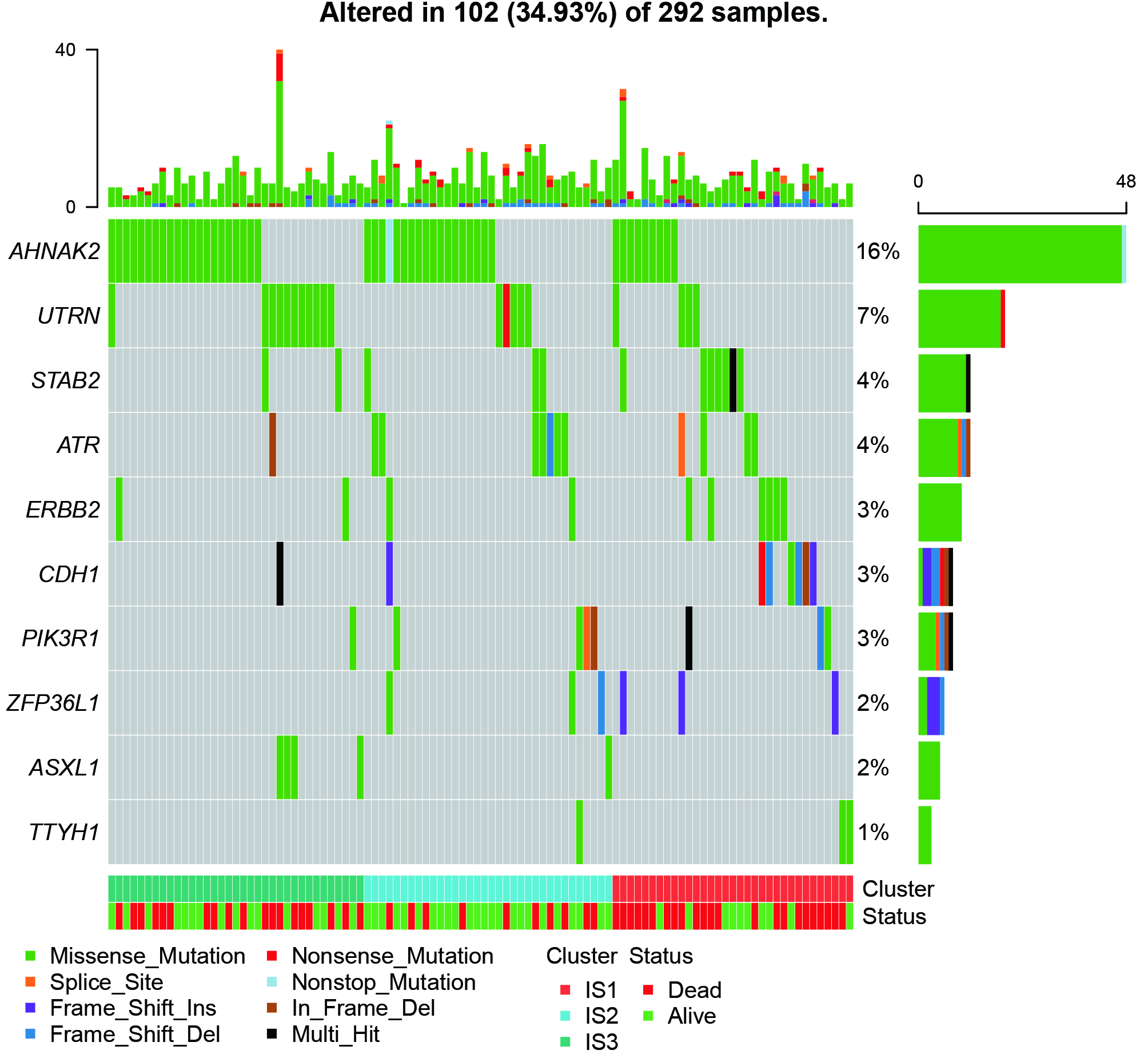

Supplement: Supplementary file 1 [file DataSheet1.ZIP › S_Fig. turquoise.tif]
